# Stiffness-Activated GEF-H1 Expression Exacerbates LPS-Induced Lung Inflammation

**DOI:** 10.1371/journal.pone.0092670

**Published:** 2014-04-16

**Authors:** Isa Mambetsariev, Yufeng Tian, Tinghuai Wu, Tera Lavoie, Julian Solway, Konstantin G. Birukov, Anna A. Birukova

**Affiliations:** Lung Injury Center, Section of Pulmonary and Critical Medicine, Department of Medicine, University of Chicago, Chicago, Illinois, United States of America; University of Kentucky, United States of America

## Abstract

Acute lung injury (ALI) is accompanied by decreased lung compliance. However, a role of tissue mechanics in modulation of inflammation remains unclear. We hypothesized that bacterial lipopolysacharide (LPS) stimulates extracellular matrix (ECM) production and vascular stiffening leading to stiffness-dependent exacerbation of endothelial cell (EC) inflammatory activation and lung barrier dysfunction. Expression of GEF-H1, ICAM-1, VCAM-1, ECM proteins fibronectin and collagen, lysyl oxidase (LOX) activity, interleukin-8 and activation of Rho signaling were analyzed in lung samples and pulmonary EC grown on soft (1.5 or 2.8 kPa) and stiff (40 kPa) substrates. LPS induced EC inflammatory activation accompanied by expression of ECM proteins, increase in LOX activity, and activation of Rho signaling. These effects were augmented in EC grown on stiff substrate. Stiffness-dependent enhancement of inflammation was associated with increased expression of Rho activator, GEF-H1. Inhibition of ECM crosslinking and stiffening by LOX suppression reduced EC inflammatory activation and GEF-H1 expression in response to LPS. *In vivo*, LOX inhibition attenuated LPS-induced expression of GEF-H1 and lung dysfunction. These findings present a novel mechanism of stiffness-dependent exacerbation of vascular inflammation and escalation of ALI via stimulation of GEF-H1 - Rho pathway. This pathway represents a fundamental mechanism of positive feedback regulation of inflammation.

## Introduction

Exacerbated inflammation and lung barrier dysfunction are hallmarks of acute respiratory distress syndrome (ARDS), a condition with dangerously high rates of morbidity and mortality. Along with acute alterations in blood-gas barrier and inflammatory activation of lung cells, lung injury also stimulates provisional extracellular matrix formation which persists during the fibroproliferative phase in ARDS. These matrices further emit signals to activate an additional inflammatory response, but may also lead to permanent matrix remodeling [1]. Indeed, activated synthesis and deposition of extracellular matrix (ECM) proteins including collagens III, IV, fibronectin and growth factors has been observed in lungs ventilated with high peak airway pressure [2] representing clinically relevant scenario of ARDS.

Due to its functional requirements, the lung is a biomechanically sensitive organ which is particularly dependent on the composition and architectural organization of ECM components. Lysyl oxidase (LOX) is an extracellular enzyme that catalyzes oxidative deamination of peptidyl lysine and hydroxylysine residues in secreted collagen precursors, and lysine residues in elastin leading to ECM fibers cross-linking. Excess LOX-dependent cross-linking contributes to excess ECM accumulation and stiffening in fibrotic diseases [3]. In turn, inhibition of LOX with inhibitory antibodies or irreversible chemical inhibitor β-aminopropyl nitrile (BAPN) reduced the cross-linking of fibrillar collagen and tissue tension which was sufficient to impede tumor progression [4], reverse established fibroblast activation and promote resolution of fibrosis [5]. These dramatic effects of stiffness modulation in pathologic conditions are due to fundamental role of cell sensing of mechanical microenvironment on cell fate and physiologic responses. It has been shown that that matrix stiffness affects cell signaling, cytoskeletal organization, levels of intercellular and intracellular force generation [6]–[8], and may even define a fate of progenitor cells directing them towards neuronal, muscle or bone lineages [9]. The relation between stiffness and modulation of inflammation is unknown.

Our recent study demonstrated significant increase in thrombin-induced actin stress fiber formation and signaling in human pulmonary endothelial cells grown on stiff (42 kPa) substrate as compared to cells grown on matrices of more physiological stiffness [10]. Several reports demonstrate that stiffness-dependent cell responses including endothelial barrier disruption, cell differentiation and cell motility are mediated by the levels of stiffness-dependent activation of small GTPase Rho [10]–[12].

Tissue inflammation resulting from bacterial infections or sepsis is triggered by Toll-like receptor (TLR)-mediated stimulation of inflammatory signaling cascades including TLR4-MyD88-IRAK-TRAF6 cascade, p38 MAPK, Erk-1,2, JNK stress kinase and NFkB activation [13]. Interestingly, these canonical inflammatory cascades may be also positively regulated by RhoA, whereas genetic model of upregulated Rho activity demonstrated sustained NFkB activation in cells and tissues [14]. Inhibition of Rho pathway attenuates LPS-induced inflammation [15], however precise mechanisms of such signaling interactions are not completely clear. Thus, existing data suggest that prolonged Rho activation induced by LPS may be implied in additional stimulation of NFkB signaling in inflamed lungs, although specific mechanisms of Rho activation by inflammatory stimuli in ALI still remain to be elucidated.

Guanine nucleotide exchange factor H1 (GEF-H1) is a Rho-specific GEF, which localizes on microtubules (MT). In MT-bound state, the guanine-exchange activity of GEF-H1 is suppressed but activated upon GEF-H1 release caused by MT disassembly [16]. We have recently reported the protective effect of MT stabilization against thrombin-induced Rho activation and barrier compromise and demonstrated the essential role of MT-associated Rho-specific nucleotide exchange factor GEF-H1 in MT-mediated regulation of Rho activity, cytoskeletal remodeling and EC permeability [17]. GEF-H1 plays a role as a mechanotransducer leading to Rho activation in endothelial cells exposed to cyclic stretch and EC barrier dysfunction [18]. In addition, we have recently shown that GEF-H1-mediated activation of Rho positively regulates LPS-induced EC barrier dysfunction *in vitro* as well as increases vascular leak and lung inflammation *in vivo*. Such disruptive effects were linked to activation of Rho signaling caused by LPS-induced MT disassembly and release of Rho-specific GEF-H1 from MTs [19].

Based on these findings, we hypothesized that LPS-induced inflammation, increased ECM synthesis and activation of Rho signaling may interplay via stiffness-dependent mechanisms. We examined LPS effects on expression of ECM proteins and ECM-modifying enzyme LOX, evaluated effects of substrate stiffness on LPS-induced EC inflammatory activation and tested role of GEF-H1 as potential mechanism of stiffness-dependent stimulation of Rho signaling and exacerbation of LPS-induced inflammation.

## Materials and Methods

### Cell culture and reagents

Human pulmonary artery endothelial cells (HPAEC) and human lung microvascular endothelial cells (HLMVEC) were obtained from Lonza (Allendale, NJ), maintained in a complete culture medium according to the manufacturer's recommendations and used for experiments at passages 5–7. Unless specified, biochemical reagents were obtained from Sigma (St. Louis, MO). TNFα was purchased from R&D Systems (Minneapolis, MN); β-aminopropyl nitrile (BAPN) was from Sigma. Antibodies to fibronectin, LOX, VCAM1, and ICAM1 were obtained from Santa Cruz Biotechnology (Santa Cruz, CA); antibpdies to phosphorylated myosin light chain phosphatase (MYPT), diphospho-myosin light chain (MLC), GEF-H1 were from Cell Signaling (Beverly, MA). All reagents for immunofluorescence were purchased from Molecular Probes (Eugene, OR). LOX activity and interleukin-8 (IL-8) production in conditioned medium was measured using LOX activity assay (AAT Bioquest, Sannyvale, CA) and IL-8 ELISA kit (R&D Systems, Minneapolis, MN), respectively, according to the manufacturers' instructions.

### Human lung slices

Human donor lungs that could not be transplanted were obtained from deceased donors through Gift of Hope/Regional Organ Bank of Illinois and were stored at 4°C for up to 2 days before use. Precision cut lung slices were obtained and cultured as previously described [20].

### Preparation of polyacrylamide (PAA) substrates for endothelial cell cultures

PAA substrates were prepared on glass coverslips with an acrylamide/bis-acrylamide ratio to obtain gels with shear elastic moduli of 1.5 kPa, 2.8 kPa, and 40 kPa and coated with collagen as characterized previously [21]. Collagen was covalently attached to the top surface of the PAA hydrogel by using the bifunctional crosslinker sulfo-SANPAH (Pierce Thermo Scientific, Rockford, IL).

### siRNA transfection

For GEF-H1 or LOX knockdown, pre-designed ON-TARGET plus SMARTpool human GEF-H1- or LOX-specific siRNA sets were ordered from Dharmacon (Lafayette, CO). Transfection of EC with siRNA was performed as previously described [18]. After 72 hrs of transfection cells were used for experiments or harvested for western blot verification of specific protein depletion. Non-specific RNA (Dharmacon) was used as a control treatment.

### Western blot

Protein extracts from lung or EC homogenates were separated by SDS-PAGE, transferred to polyvinylidene difluoride membranes, and the membranes were incubated with specific antibodies of interest. Equal protein loading was verified by reprobing membranes with β-actin antibodies. Immunoreactive proteins were detected with the enhanced chemiluminescent detection system according to the manufacturer's protocol (Amersham, Little Chalfont, UK).

### Immunofluorescence staining

Endothelial monolayers plated on glass cover slips were subjected to immunofluorescence staining as described previously [18]. Slides were analyzed using Nikon video-imaging system (Nikon Instech, Tokyo, Japan). Images were processed using Adobe Photoshop 7.0 software (Adobe Systems, San Jose, CA).

### Quantitative reverse-transcription polymerase chain reaction (qRT-PCR)

Reverse transcription (RT) was performed with 1 µg of total RNA isolated from EC. RT-PCR reactions were performed as previously described and gene expression fold changes were calculated according to the ΔΔCt method [22]. The following primers were used: fibronectin: *5′-CTTTGGTGCAGCACAACTTC-3′* (forward) and *5′-TGGAATTTCCTCCTCGAGTC-3′* (reverse); collagen type I A1: *5′-AAGAGGAAGGCCAAGTCGAG-3′* (forward) and *5′-AGATCACGTCATCGACAAC-3′* (reverse); *LOX*: *5′-CATCATGCGTATGCCTCAG-3′* (forward) and *5′-TTCCCACTTCAGAACACCAG-3′* (reverse); GAPDH: *5′-AGGTGAAGGTCGGAGTCAAC-3′* (forward) and *5′-AGTTGAGGTCAATGAAGGGG-3′* (reverse).

### Animal studies

All experimental protocols involving the use of animals were approved by the University of Chicago Institutional Animal Care & Use Committee for the humane treatment of experimental animals. C57BL/6J mice were randomized to concurrently receive sterile saline solution or BAPN (100 mg/kg) by intraperitoneal injection the day before intratracheal LPS administration (0.83 mg/kg; *Escherichia coli* O55:B5) and then injected daily. After 72 hours of LPS challenge, animals were sacrificed by exsanguination under anesthesia. Measurements of cell count, protein concentration, Evans blue extravasation and histological assessment of lung injury were conducted as described [23].

### Statistical analysis

Results are expressed as means ±SD of three to eight independent experiments. Stimulated samples were compared to controls by unpaired Student's t-test. For multiple-group comparisons, one-way ANOVA and Tukey's post hoc multiple-comparison test were used. P<0.05 was considered statistically significant.

## Results

### LPS stimulates Rho signaling, expression of adhesion molecules and ECM proteins in endothelial cells

**Figure 1:**
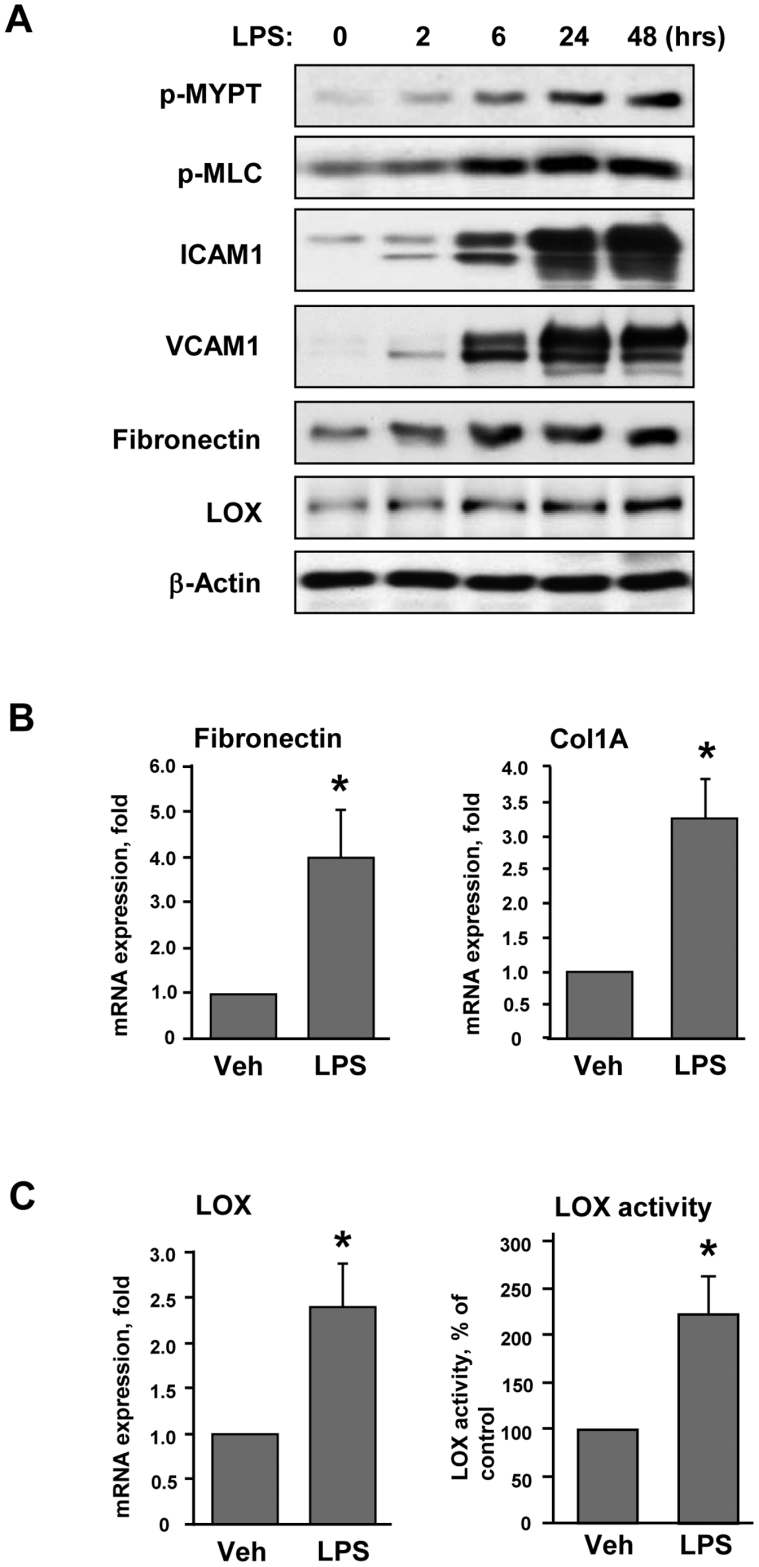
Characterization of LPS-induced activation of pulmonary EC. Human pulmonary EC were grown on 2.8(200 ng/ml). **A** - Time-dependent phosphorylation of MYPT and MLC and expression of ICAM-1, VCAM-1, fibronectin and LOX was determined by western blot analysis. Equal protein loading was confirmed by membrane re-probing with β-actin antibody. **B** – Analysis of fibronectin and collagen 1A (Col1A) mRNA levels after 12-hr LPS treatment was performed by RT-PCR; **C** – Analysis of LOX mRNA levels and LOX activity in conditioned medium from control and LPS-stimulated (12 hrs) cells; *p<0.05.

Pulmonary EC grown on polyacrylamide hydrogels of physiologically relevant stiffness (2.8 kPa) were stimulated with LPS. LPS induced time-dependent phosphorylation of MYPT and MLC (**Figure 1A**). Site specific phosphorylation of MYPT at Thr^850^ and corresponding increase in phospho-MLC levels is a well recognized parameter of activated Rho signaling. Delayed activation of Rho signaling was accompanied by LPS-induced upregulation of the markers of EC inflammatory activation ICAM-1 and VCAM-1. LPS stimulated expression of extracellular matrix proteins fibronectin (FN) and lysyl oxidase (LOX), the enzyme involved in ECM crosslinking and stiffening of extracellular matrix [3]. LPS stimulation increased mRNA levels of FN and Collagen-1A detected by RT-PCR analysis (**Figure 1B**). LPS also increased mRNA levels as well as enzymatic activity of LOX in the conditioned media from LPS-stimulated endothelial cells (**Figure 1C**).

### LOX inhibition attenuates LPS-induced inflammatory activation

**Figure 2:**
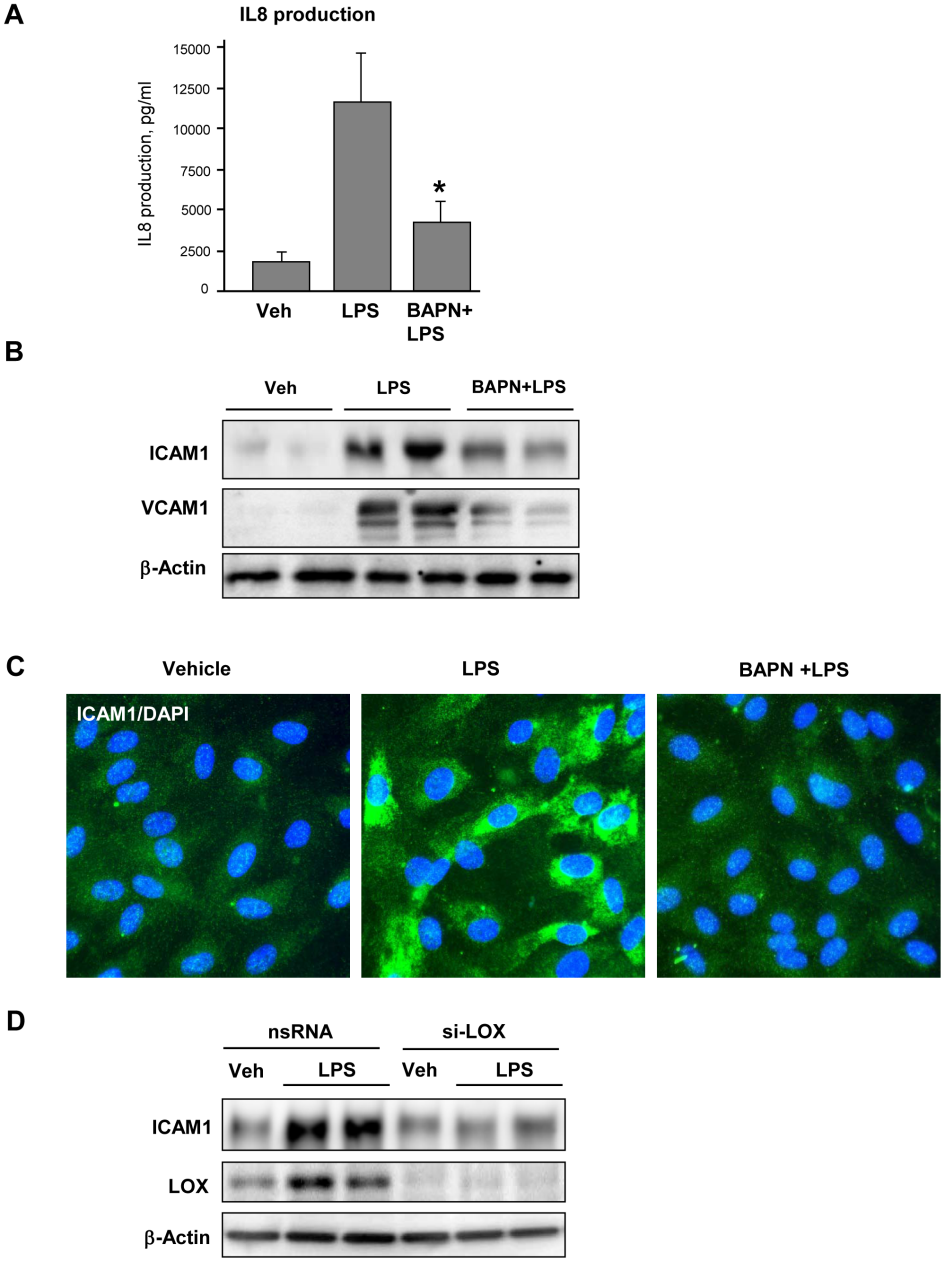
Effect of LOX inhibition on LPS-induced EC inflammatory activation. Human pulmonary EC grown on 2.8(300 µM), and then stimulated with LPS (200 ng/ml) for 48 hrs with or without BAPN. **A** – IL-8 production by EC stimulated with or without BAPN was evaluated in conditioned medium by ELISA assay; *P<0.05. **B** – Expression of ICAM-1 and VCAM-1 was determined by western blot analysis with specific antibodies. **C** – ICAM-1 expression was examined by immunofluorescence staining of stimulated EC using ICAM-1 antibody (green). Counterstaining with DAPI (blue) was used to visualize cell nuclei. **D** – HPAEC were transfected with non-specific (nsRNA) or LOX-specific siRNA (si-LOX). ICAM-1 expression was determined by western blot. Equal protein loading was confirmed by membrane re-probing with β-actin antibody.

In these experiments, pulmonary EC were cultured on 2.8 kPa polyacrylamide hydrogels for 48 hrs in the presence of LPS with or without LOX irreversible inhibitor BAPN. Co-incubation with BAPN attenuated LPS-induced IL-8 production and expression of ICAM-1 and VCAM-1 (**Figure 2AB**). Inhibitory effect of BAPN was also confirmed by attenuation of LPS-induced ICAM-1 expression detected by immunofluorescence staining of EC monolayers with ICAM-1 antibody (**Figure 2C**). Attenuation of LPS-induced ICAM-1 expression was also achieved by LOX knockdown using siRNA approach (**Figure 2D**).

### Substrate mechanics modulates LPS-induced EC inflammatory activation

**Figure 3:**
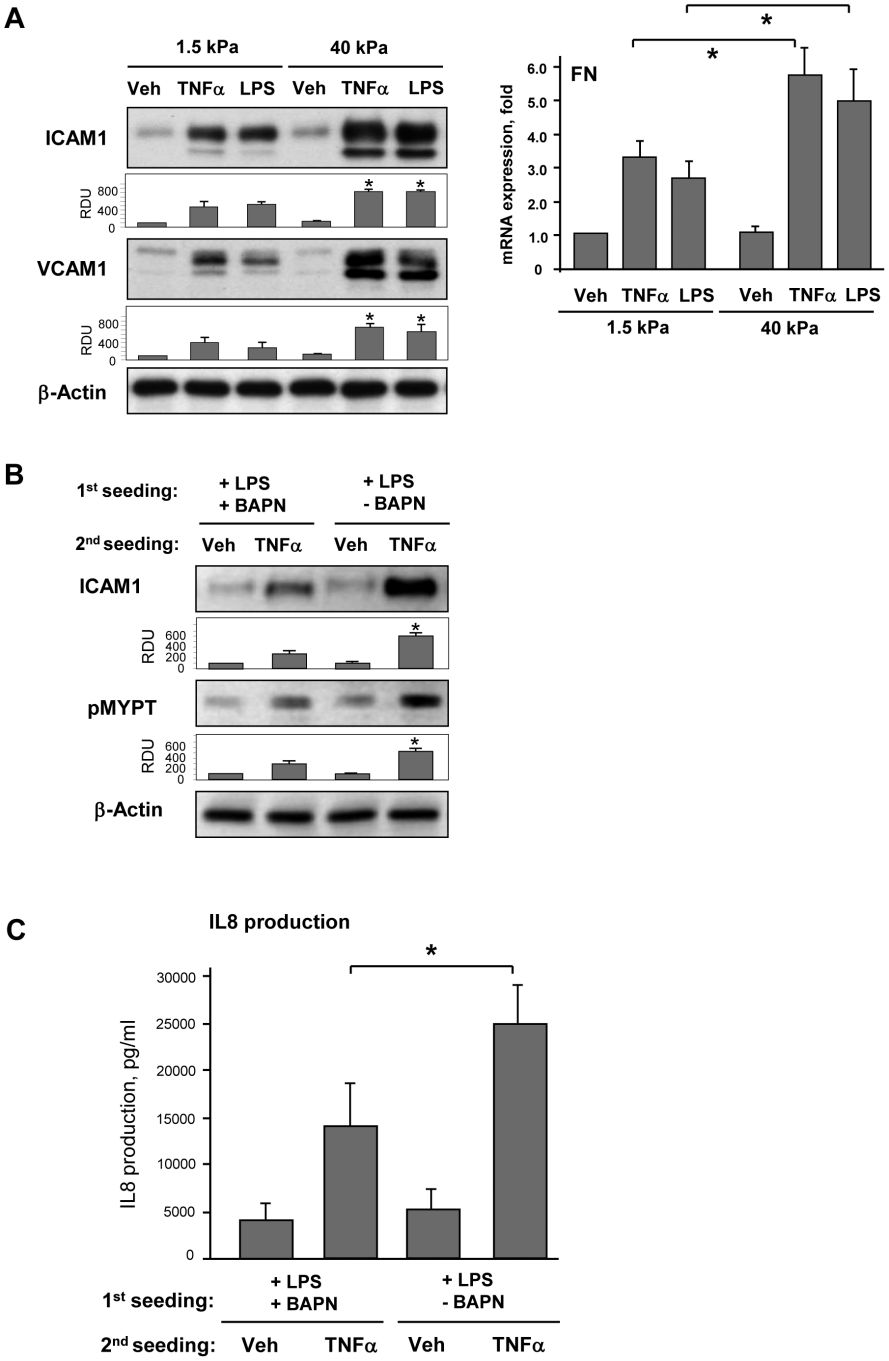
Effect of substrate stiffness and LOX inhibition on LPS-induced EC activation. **A** - Pulmonary EC were grown on polyacrylamide gels of different stiffness (1.5 kPa, and 40 kPa) and treated with TNFα (2 ng/ml) or LPS (200 ng/ml) for 6 hrs. **Left panel**: Expression of ICAM-1 and VCAM-1 was determined by western blot analysis. Equal protein loading was confirmed by membrane re-probing with β-actin antibody. Bar graphs depict the quantitative analysis of western blot densitometry data; *P<0.05 vs. 1.5 kPa; n = 4. **Right panel**: Analysis of fibronectin mRNA levels after 12-hr treatment with LPS or TNFα was performed by RT-PCR; *p<0.05. **B** – Pulmonary EC were cultured on 2.8 kPa substrates for 3 days in the presence of LPS (200 ng/ml) with or without BAPN (300 µM). After cell detachment, fresh EC were plated on deposited extracellular matrix and stimulated with TNFα (2 ng/ml, 6 hrs). ICAM-1 expression and MYPT phosphorylation was analyzed by western blot. Equal protein loading was confirmed by membrane re-probing with β-actin antibody. Bar graphs depict the quantitative analysis of western blot densitometry data; *P<0.05 vs. LPS+BAPN treatment; n = 4. **C** - IL-8 production in response to TNFα (2 ng/ml, 6 hrs) was evaluated by ELISA assay. *P<0.05.

**Figure 4:**
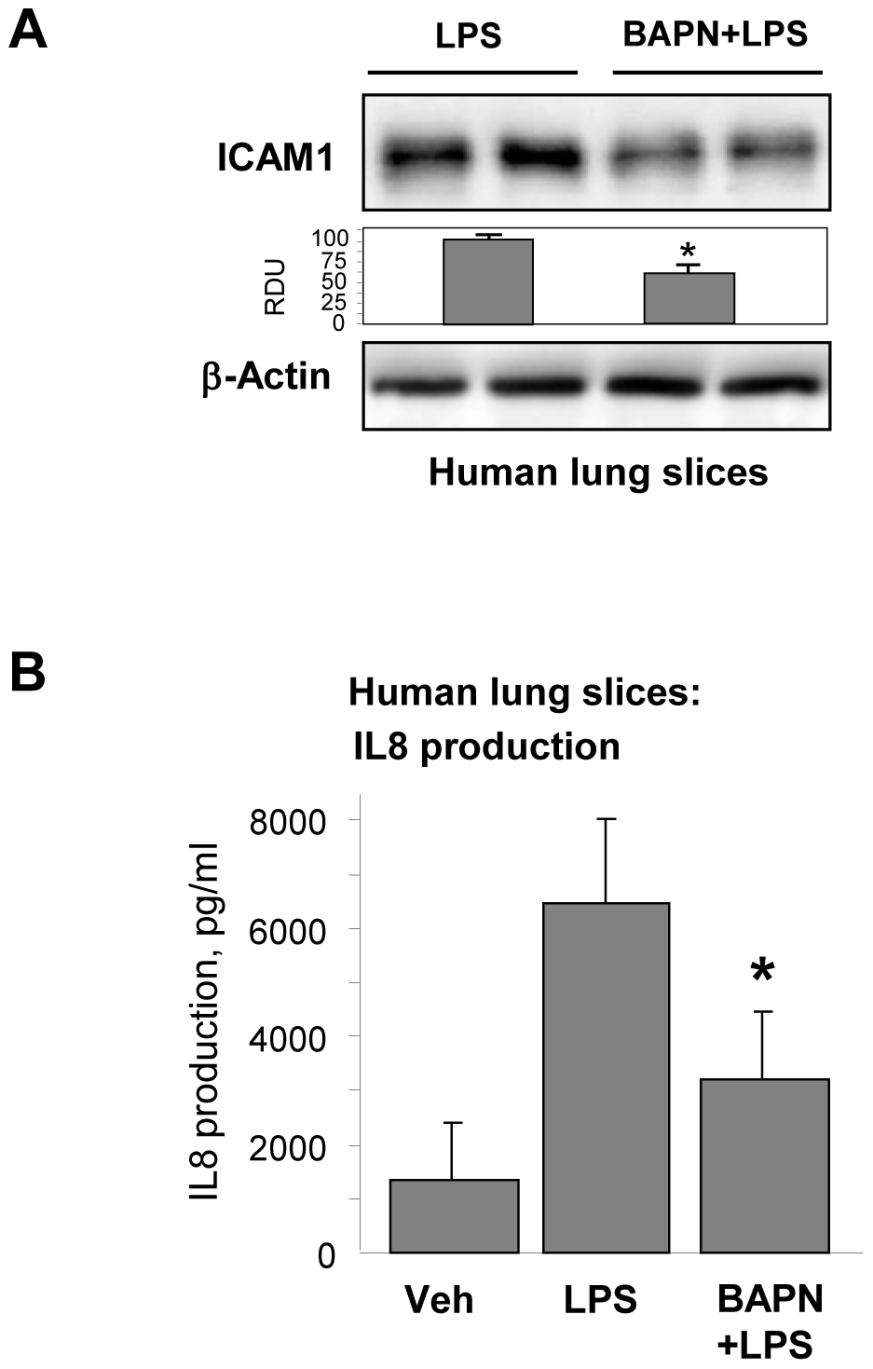
Effect of LOX inhibition on LPS-induced ICAM-1 expression and IL-8 production in human lung slices. Human lung slices were cultured during 48(200 ng/ml) with or without BAPN (300 µM). **A** - ICAM-1 expression was analyzed by western blot. Equal protein loading was confirmed by membrane re-probing with β-actin antibody. Bar graphs depict the quantitative analysis of western blot densitometry data; *P<0.05 vs. LPS; n = 4. **B** - IL-8 production was evaluated by ELISA assay of conditioned medium. *P<0.05.

Effects of substrate stiffness on inflammatory activation were tested directly in pulmonary EC grown on soft (1.5 kPa) or stiff (40 kPa) polyacrylamide hydrogels and stimulated with LPS or another ALI-relevant inflammatory mediator, TNFα for 6 hrs. Both mediators induced significantly higher levels of ICAM-1 and VCAM-1 protein expression and increased FN mRNA expression by EC grown on stiffer substrate (**Figure 3A**). Next experiments were performed to examine a role of BAPN-mediated modification of natural ECM deposited by cultured EC on cell inflammatory response. LPS-challenged pulmonary EC with or without BAPN treatment were cultured for 3 days on hydrogels (2.8 kPa) to allow for ECM deposition. After cell monolayer detachment by sterile EGTA and washing step, fresh pulmonary EC were plated on pre-deposited matrix. After cell attachment and spreading, EC cultures were stimulated with TNFα for 6 hrs. Cells on matrix produced by LPS-stimulated EC showed increased ICAM-1 expression and MYPT phosphorylation in response to TNFα as compared to cells grown on matrices produced under LPS+BAPN conditions (**Figure 3B**). EC seeded on the matrix deposited by LPS-stimulated cells also produced higher levels of IL-8 upon stimulation with TNFα in comparison to their counterparts grown on ECM deposited by cells treated with LPS and BAPN (**Figure 3C**). Precision cut human lung slices were further used to evaluate effects of BAPN on inflammatory activation in the lung tissue. Lung samples were cultured for 48 hrs with LPS, with or without BAPN. BAPN co-treatment significantly attenuated LPS-induced ICAM1 expression and IL-8 levels in conditioned medium (**Figure 4**).

### LPS-induced GEF-H1 expression by pulmonary EC is stiffness dependent

**Figure 5:**
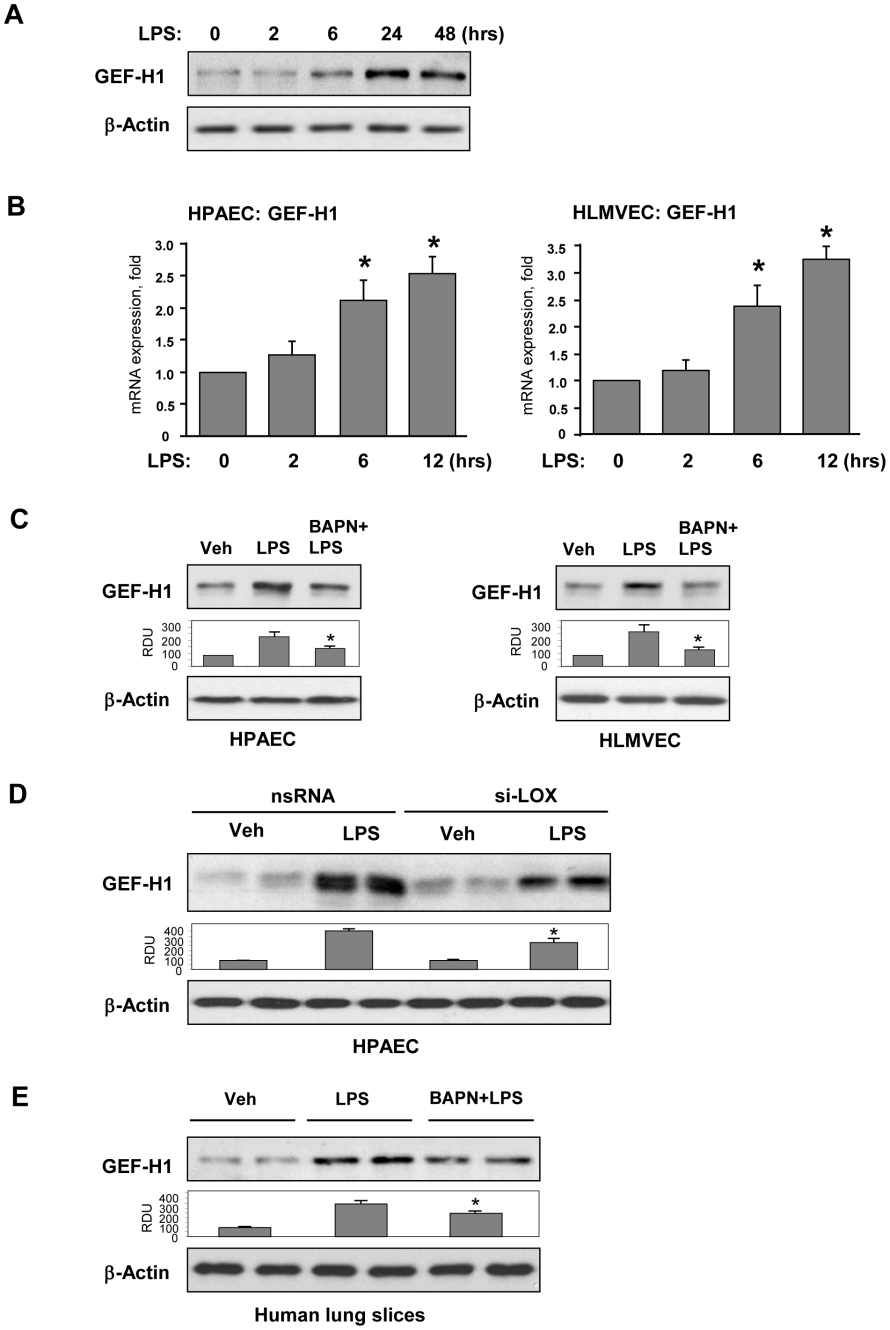
Characterization of LPS-induced expression of GEF-H1. Human pulmonary EC were on 2.8(200 ng/ml). **A** - Time-dependent expression of GEF-H1 was determined by western blot analysis. Equal protein loading was confirmed by membrane re-probing with β-actin antibody. **B** – Time-dependent analysis of LPS-induced GEF-H1 mRNA expression in human lung macrovascular EC (left panel) and microvascular EC (right panel) was performed by RT-PCR; *p<0.05; **C** – Human pulmonary macrovascular EC (left panel) or microvascular EC (right panel) grown on 2.8 kPa substrate were treated for 24 hrs with vehicle or BAPN (300 µM), and then stimulated with LPS (200 ng/ml) with or without BAPN. After 48 hrs, expression of GEF-H1 was determined by western blot analysis. Bar graphs depict the quantitative analysis of western blot densitometry data; *P<0.05 vs. LPS; n = 3. **D** – Effect of si-RNA induced LOX knockdown (72 hrs) on LPS-induced GEF-H1 expression. Bar graphs depict the quantitative analysis of western blot densitometry data; *P<0.05 vs. non-specific RNA; n = 3. **E** – Human lung slices were cultured during 48 hrs in the presence of LPS (200 ng/ml) with or without BAPN (300 µM). GEF-H1 expression was analyzed by Western blot. Equal protein loading was confirmed by membrane re-probing with β-actin antibody. Bar graphs depict the quantitative analysis of western blot densitometry data; *P<0.05 vs. LPS; n = 4.

Because LPS stimulated Rho signaling and stiffness-dependent EC inflammatory activation, we next tested whether these events can be linked via GEF-H1. LPS-stimulation of pulmonary EC grown on 2.8 kPa substrate markedly increased GEF-H1 protein expression after 24–48 hrs (**Figure 5A**) and GEF-H1 mRNA levels in human lung macrovascular and microvascular EC after 6–12 hrs of LPS treatment (**Figure 5B**). Western blot analysis of EC plated on 2.8 kPa hydrogels and stimulated with LPS or LPS+BAPN for 48 hrs showed that pharmacologic LOX inhibition significantly attenuated LPS-induced GEF-H1 expression in both cell types (**Figure 5C**). This finding was further confirmed by LOX molecular inhibition using siRNA approach (**Figure 5D**). LOX inhibition also attenuated LPS-induced GEF-H1 expression in the cultured lung slices (**Figure 5E**).

**Figure 6:**
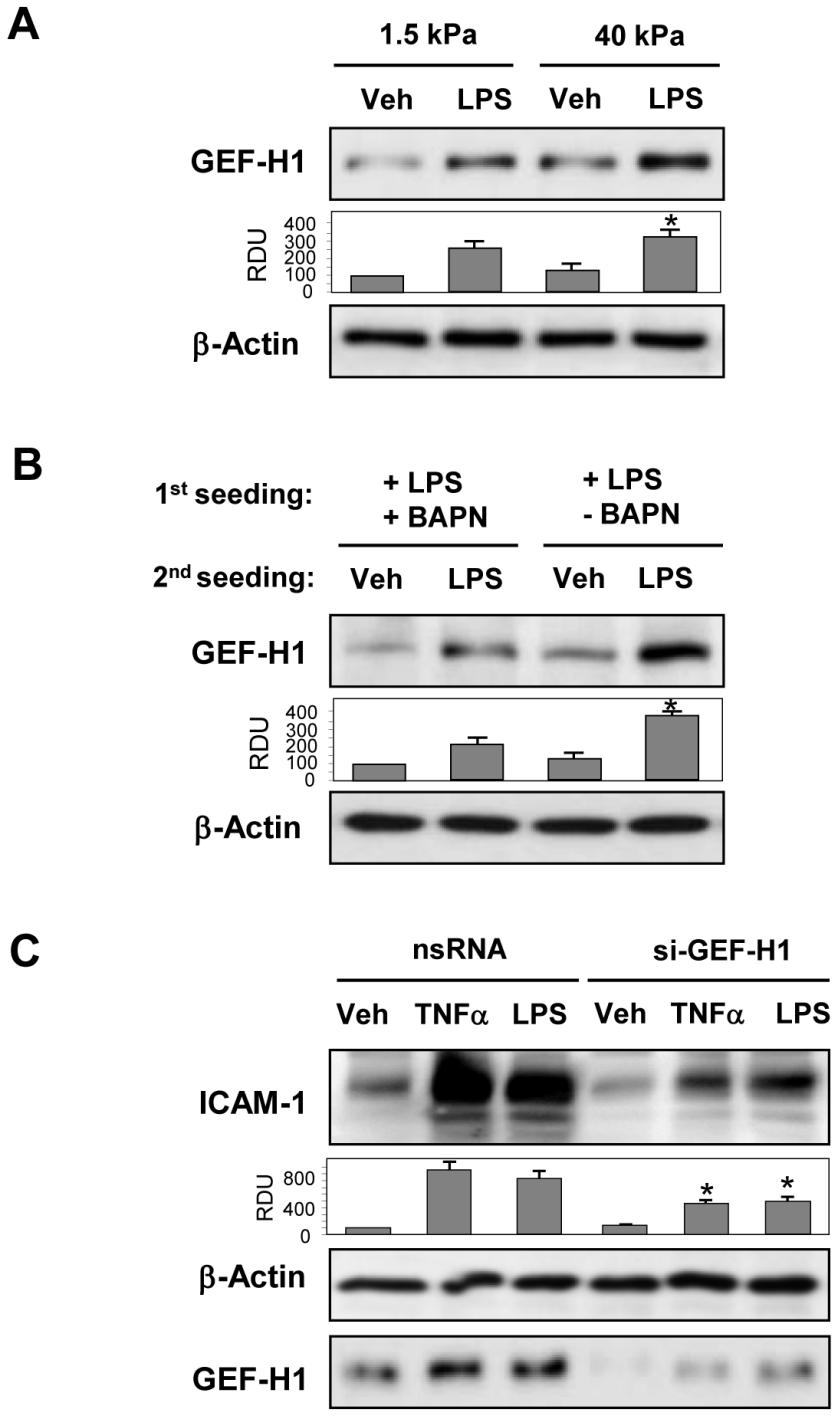
Effect of substrate stiffness and LOX inhibition on LPS-induced GEF-H1 expression. **A** - Pulmonary EC were grown on polyacrylamide gels of different stiffness (1.5 kPa, and 40 kPa) and treated with LPS (200 ng/ml) for 6 hrs. GEF-H1 expression determined by western blot analysis. Bar graphs depict the quantitative analysis of western blot densitometry data; *P<0.05 vs. 1.5 kPa; n = 4. **B** – Pulmonary EC were cultured on 2.8 kPa substrates for 3 days in the presence of LPS (200 ng/ml) with or without BAPN (300 µM). After cell detachment, fresh EC were plated on deposited extracellular matrix and stimulated with LPS (200 ng/ml, 6 hrs). GEF-H1 expression was analyzed by western blot. Bar graphs depict the quantitative analysis of western blot densitometry data; *P<0.05 vs. LPS+BAPN treatment; n = 4. **C** – Pulmonary EC grown on 2.8 kPa substrates and treated with nonspecific or GEF-H1-specific siRNA for 72 hrs were stimulated with TNFα (2 ng/ml) or LPS (200 ng/ml) for 6 hrs, and ICAM-1 expression was analyzed by western blot. GEF-H1 depletion was confirmed by membrane reprobing with GEF-H1 antibody. Equal protein loading was confirmed by membrane re-probing with β-actin antibody. Bar graphs depict the quantitative analysis of western blot densitometry data; *P<0.05 vs. non-specific RNA; n = 3.

Similarly to approach described above, effects of substrate stiffness on LPS-induced GEF-H1 expression were tested directly in EC cultured for 48 hrs on 1.5 kPa or 40 kPa hydrogels. LPS-induced GEF-H1 expression was enhanced in the cells grown on 40 kPa substrate (**Figure 6A**). Of note, basal GEF-H1 expression level was also higher in cells grown on 40 kPa substrate.

Effect of BAPN-mediated modification of natural ECM on LPS-induced expression of GEF-H1 was examined using cell re-seeding approach described above. LPS-challenged EC monolayers with or without BAPN co-treatment were cultured for 3 days on 2.8 kPa substrates. After cell detachment and cell re-seeding step, GEF-H1 expression was evaluated in fresh pulmonary EC stimulated with LPS for 6 hrs. BAPN inhibited LPS-induced GEF-H1 expression by EC (**Figure 6B**). We observed a trend to a slight decrease in GEF-H1 expression levels in EC plated on the matrix pre-deposited by cells grown with BAPN inhibitor, but these differences did not reach statistical significance.

To test whether induction of GEF-H1 contributes to endothelial inflammatory activation, cells were treated with control or GEF-H1 specific siRNA follwed by stimulation with inflammatory agonists. GEF-H1 depletion attenuated ICAM-1 expression induced by LPS or TNFα (**Figure 6C**).

### LOX inhibitor attenuates LPS-induced lung inflammation, vascular leak and neutrophil infiltration

**Figure 7:**
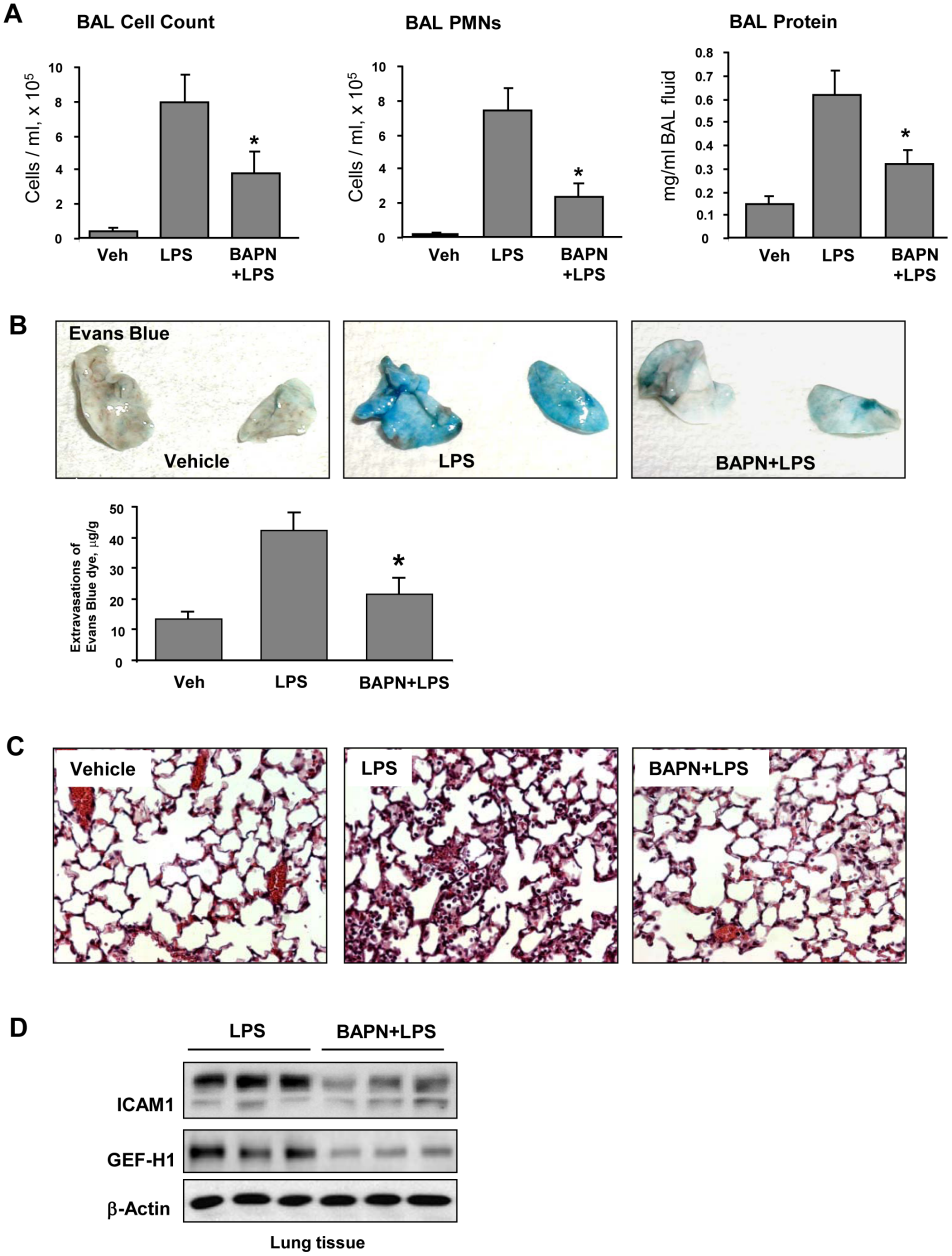
Effect of LOX inhibitor on LPS-induced lung inflammation and barrier dysfunction. C57BL/6J mice were challenged with vehicle or LPS (0.63 mg/kg, i/t) with or without BAPN treatment (100 mg/kg). Control animals were treated with sterile saline solution. **A** – Total cell count, PMN cell count and protein concentration were determined in bronchoalveolar lavage fluid collected 48 hrs after treatments. **B** - Evans blue dye (30 ml/kg, i/v) was injected 2 hr before termination of the experiment. Lung vascular permeability was assessed by Evans blue accumulation in the lung tissue. The quantitative analysis of Evans blue labeled albumin extravasation was performed by spectrophotometric analysis of Evans blue extracted from the lung tissue samples; *p<0.05. **C** – Histological analysis of lung tissue by hematoxilin & eosin staining (×40 magnification); **D** - Expression of ICAM-1 and GEF-H1 in lung tissue samples evaluated by western blot analysis. Equal protein loading was confirmed by membrane re-probing with β-actin antibody.

Intratracheal administration of LPS significantly increased protein content, total and neutrophil cell count in BAL samples (**Figure 7A**). BAPN treatment significantly attenuated parameters of LPS-induced lung dysfunction. Effects of LOX inhibition on LPS-induced lung vascular leak were detected by Evans blue dye accumulation in the lung parenchyma. Images of lung preparations show LPS-induced Evans blue accumulation in the lung tissue which was attenuated by BAPN treatment (**Figure 7B**). Quantitative analysis of Evans blue-labeled albumin extracted from lungs further confirmed these results (**Figure 7B** **, lower panel**). Histological analysis of lung tissue sections stained with hematoxylin and eosin revealed that in contrast to control animals, intratracheal LPS injection induced neutrophil infiltration in the lung parenchyma and accumulation of protein-rich fluid in alveolar space indicative of alveolar-capillary barrier dysfunction. In consistence with results of BAL analysis and Evans Blue extravasation assay, BAPN administration suppressed LPS effects (**Figure 7C**). BAPN treatment also decreased protein levels of ICAM-1 and GEF-H1 in the lungs from LPS-treated mice (**Figure 7D**).

## Discussion

ALI/ARDS is associated with decreased compliance suggesting the stiffening of lung tissue, but the role of alterations in lung tissue mechanical properties in propagation of lung inflammation remains unclear. This study is the first report of stiffness-dependent enhancement of lung vascular endothelial inflammation induced by bacterial compounds. This conclusion is supported by increased expression and deposition of fibronectin and collagen-1 as well as by upregulation of LOX in LPS-activated pulmonary EC, cultured lung slices and in lung tissues of LPS-challenged mice. The responses to LPS were observed both, in the pulmonary EC culture and in human lung slices incubated *ex vivo*. EC inflammatory activation under these conditions was manifested by IL-8 production and expression of ICAM-1 and VCAM-1, the EC surface adhesion molecules essential for leukocyte adhesion [24].

The basal membrane in the vascular capillaries typically contains collagen IV which forms compliant meshwork [25]. LPS-induced expression of collagen-I and LOX by LPS-stimulated EC shown in this study leads to formation of cross-linked fibers with increased mechanical stiffness [25] and thus suggests significant alterations in EC mechanical microenvironment. Experiments with seeding pulmonary EC on matrices pre-deposited by LPS-stimulated EC in the presence or absence of BAPN were designed to test effects of LOX-mediated crosslinking which alters ECM mechanical properties on agonist-induced EC inflammatory activation monitored by ICAM-1, VCAM-1 and IL-8 upregulation. By inhibiting LOX enzymatic activity, BAPN prevents collagen crosslinking thus reducing stiffness of synthesized matrix [4], [25]. Alternatively LOX was inhibited using siRNA-induced knockdown. Inhibition of LOX function attenuated pro-inflammatory potential of ECM produced by LPS-stimulated endothelium. Obtained results strongly suggest that LPS activation of pulmonary EC can indeed define the mechanical properties of newly synthesized ECM leading to the ECM stiffening and further stimulation of EC inflammatory responses. Previous report supports this notion and demonstrates formation of highly stretched, stiffer matrix deposited by stromal cells activated by tumor-secreted soluble factors, which then contributes to tumorigenesis [26]. However, further studies are needed to more precisely evaluate the mechanical properties of endogenous matrix deposited by EC in inflammatory conditions.

The role of substrate stiffness on EC activation was directly tested in experiments using EC grown on polyacrylamide hydrogels of defined stiffness. We compared cells grown on substrates of physiological stiffness range (1.5 kPa) and elevated stiffness (40 kPa). This stiffness range was detected in perivascular regions of LPS-challenged lungs by AFM measurements in precision cut lung slices (K. Birukov, M. Allen, unpublished data). LPS caused more pronounced ICAM-1 expression and IL-8 production in EC grown on stiff substrate, thus supporting the stiffness dependent mechanism of inflammatory propagation.

In agreement with proposed hypothesis considering the role of lung stiffening in exacerbation of LPS-induced inflammation, the pretreatment of mice with BAPN prior to intratracheal LPS administration significantly attenuated parameters of lung injury and inflammation. Previous study shows the two-fold increase in LOX activity in the lungs of LPS-stimulated mice [27]. Since BAPN did not alter collagen expression levels in lung *in vivo* [27], these data support the mechanism of BAPN inhibitory effects on inflammatory activation in vitro and in vivo due to effects on ECM crosslinking and stiffness, rather than alterations in ECM expression. Chronic BAPN pretreatment (2 weeks) has been beneficial for attenuation of tumor progression [4], but such protocol also caused a loss of the fine network of collagen I, III, IV, VI-containing fibrils and disruption of the well-defined borders of alveolar structures [27]. The shorter BAPN pretreatment prior to LPS challenge used in this study was selected to inhibit immediate effects of LPS challenge without affecting the preexisting status of lung tissue. Further technological developments such as AFM methods will be essential to precisely characterize local changes in ECM mechanics and tissue stiffness within distinct regions of the lung or lung microvasculature. In addition to stiffness-dependent modulation of lung endothelial inflammatory response, we do not exclude the impact of local micromechanics changes as result of provisional matrix deposition in the course of ALI on the inflammatory status of other cells such as alveolar epithelium, resident macrophages and fibroblasts. We also cannot exclude additional mechanisms of ALI exacerbation by ECM stiffening such as release of ECM-associated bioactive molecules.

Previous studies by our group and others showed requirement of the Rho signaling for the full activation of LPS-induced lung inflammation [15], [19], [28]–[30]. We proposed an upstream mechanism of LPS-induced GEF-H1 activation via LPS- and ROS-dependent disassembly of microtubules leading to activation of Rho-specific GEF-H1 guanine nucleotide exchange activity [19]. Rho is a downstream target of GEF-H1 and activates its effector, Rho-kinase, leading to increased phosphorylation of MLC, actomyosin contraction and barrier dysfunction. Rho and Rho kinase my directly stimulate NFkB cascade via yet to be identified mechanisms [14], [28]. Such Rho-dependent stimulation of NFkB cascade leads to increased expression of its downstream targets, the EC inflammation markers ICAM1, VCAM and IL-8 [19], [30], [31]. These data emphasize the role of Rho signaling in modulation of inflammation.

Besides pro-fibrotic mechanisms including matrix deposition at later phase of inflammation, local tissue stiffness in the inflamed organ may change due to other factors, for example tissue swelling. This event develops relatively fast and is consistent with the time frame of GEF-H1 activation, Rho signaling and expression of inflammatory markers including ECM protein fibronectin described in our study, which exacerbate cell inflammatory response to pro-inflammatory mediator. Stiffness effects on GEF-H1 expression were seen as early as 6 hrs after cell plating (Figure 6A), and were consistent with stiffness-dependent effects on TNFα-induced upregulation of ICAM1 and VCAM1. Because experimental models recapitulating tissue swelling *in vitro* are currently unavailable, we focused on ECM deposition by cultured EC and cell plating on polyacrylamide hydrogels of different stiffness. Our other unpublished study using atomic force microscopy probing the crossection area of lung microvessel in precision cut live lung slices shows five-fold increase of local stiffness in the microvascular region after 48 hrs of LPS intratracheal instillation in mouse lungs. These findings support our hypothesis about local micromechanical changes in lung microvasculature of inflamed lungs.

One potential mechanism of stiffness-dependent regulation of inflammation may include activation of Rho-Rho kinase signaling which can be activated by cyclic stretch or pulling single cells by micropipette or electromagnetic field applied to cell-attached magnetic beads [32], [33]. The magnitude of applied cyclic stretch determines the magnitude of Rho activation and CS-dependent enhancement of agonist-induced endothelial permeability [34]. Recent reports show positive correlation between increasing substrate stiffness and Rho activation [10]–[12]. Increased stiffness also augmented agonist-induced activation of Rho pathway and EC barrier disruption [10]. On the other hand, Rho signaling is essential for full activation of inflammation induced by soluble mediators [35], [36]. Taking into account the LPS- and stiffness-induced stimulation of the Rho pathway, we tested the potential enhancement of LPS-induced inflammatory signaling by stiffness-dependent Rho mechanism. GEF-H1 is activated by physical stimuli, including mechanical force and hyperosmolarity [12], [18], [33], [37]. Alterations in microtubule dynamics caused by pathologic mechanical forces, edemagenic mediators or LPS lead to GEF-H1 release from microtubules and activation of Rho pathway. Partial disassembly of microtubules and release of microtubule associated GEF-H1 was also reported as a mechanism of the LPS-induced activation of Rho and EC barrier dysfunction [19]. This study shows the LPS-induced activation of GEF-H1 expression which was further increased by high substrate stiffness and was therefore linked to the stiffness-dependent enhancement of ICAM-1 and VCAM-1 expression. Since LPS-stimulated inflammatory signaling was abolished by GEF-H1 knockdown, these data strongly suggest the GEF-H1 - Rho mechanism of stiffness dependent EC inflammatory activation and exacerbation of LPS-induced lung injury.

**Figure 8:**
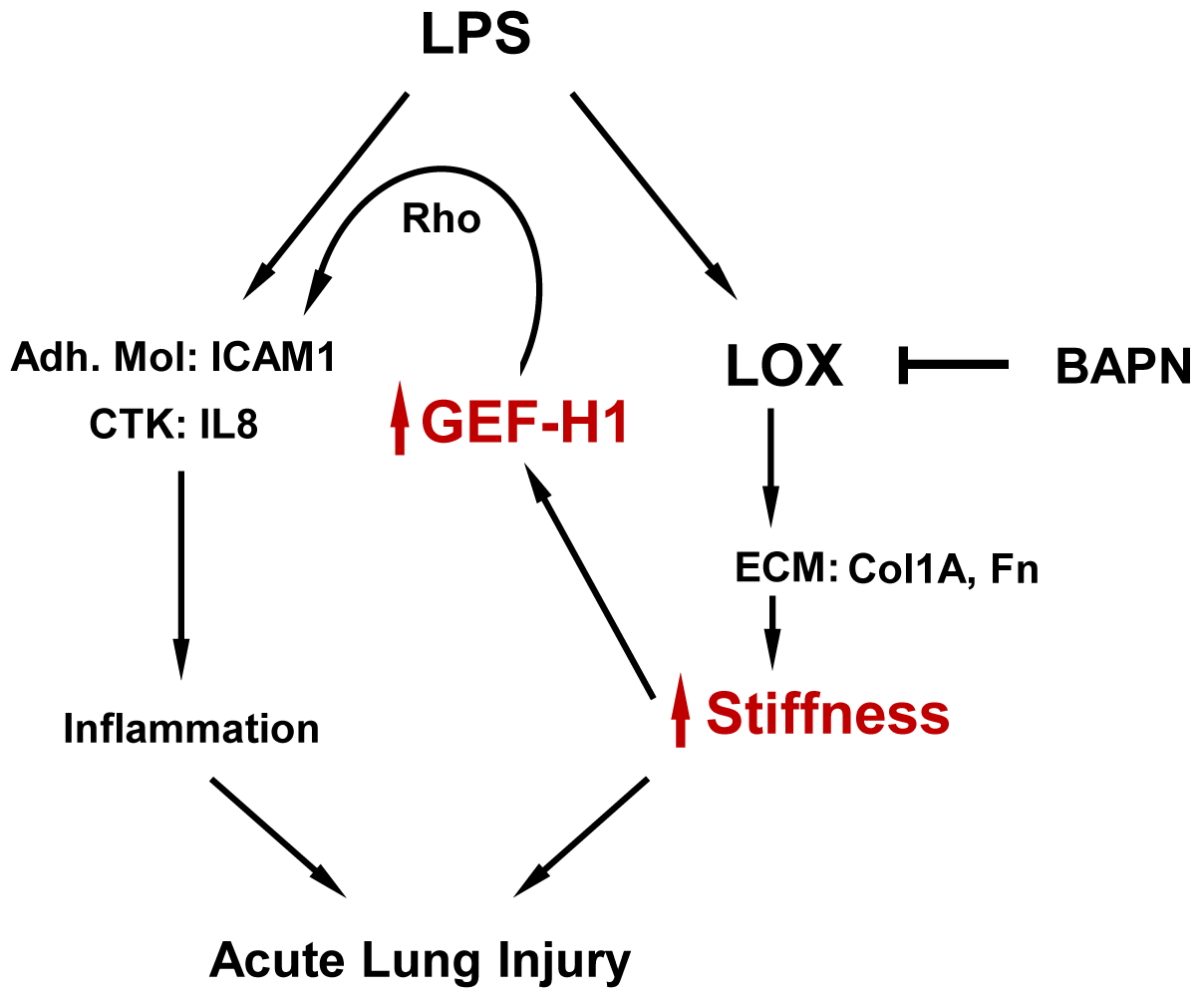
Enhancement of LPS-induced inflammation and acute lung injury via stiffness-dependent stimulation of GEF-H1. LPS or other inflammatory interventions activate inflammatory cascades in endothelial cells manifested by cytokine production and expression of leukocyte adhesion molecules. In addition, LPS stimulates expression of ECM proteins and LOX leading to local tissue stiffening and stiffness-induced expression of GEF-H1. GEF-H1 - Rho signaling pathway then provides a positive feedback mechanism leading to escalation of vascular inflammation and acute lung injury.

Based on these data, we propose that GEF-H1 plays an essential role in stiffness-dependent enhancement of LPS-induced inflammation and forms a positive feedback loop of inflammation (**Figure 8**). Via stimulation of fibronectin, collagen-I and LOX expression, LPS induces ECM deposition and crosslinking leading to further stiffening of ECM. Increased ECM stiffness in turn stimulates Rho pathway leading to exacerbation of ICAM-1, VCAM-1 and IL-8 expression by pulmonary EC. These events promote neutrophil infiltration into inflamed lungs and exacerbate ongoing lung inflammation and barrier dysfunction. The findings from this study reflect a novel mechanism of stiffness-dependent stimulation of GEF-H1 - Rho pathway leading to exacerbation of vascular inflammation and escalation of ALI. This pathway may be a fundamental mechanism of positive feedback regulation of inflammation in other organs and tissues. Thus, inhibition of GEF-H1 may be considered as approach for intervention of this vicious circle of inflammation propagation. Strategies aimed at GEF-H1 inhibition may abolish the exacerbation of ongoing inflammation and initiate the recovery phase.
